# An Insight Into the Intestinal Web of Mucosal Immunity, Microbiota, and Diet in Inflammation

**DOI:** 10.3389/fimmu.2018.02617

**Published:** 2018-11-20

**Authors:** Yu Anne Yap, Eliana Mariño

**Affiliations:** Faculty of Medicine, Nursing and Health Sciences, School of Biomedical Sciences, Monash University, Clayton, VIC, Australia

**Keywords:** mucosal immunity, gut microbiota, SCFAs, diet, IELs, autoimmunity, diabetes, infections

## Abstract

The rising global incidence of autoimmune and inflammatory conditions can be attributed to changes in the large portion of the immune system that belongs to our gastrointestinal tract (GI). The intestinal immune system serves as a gatekeeper to prevent pathogenic invasions and to preserve a healthier gut microbiota. The gut microbiota has been increasingly studied as a fundamental contributor to the state of health and disease. From food fermentation, the gut microbiota releases metabolites or short chain fatty acids (SCFAs), which have anti-inflammatory properties and preserve gut homeostasis. Immune responses against food and microbial antigens can cause inflammatory disorders such as inflammatory bowel disease (IBD) and celiac disease. As such, many autoimmune and inflammatory diseases also have a “gut origin”. A large body of evidence in recent years by ourselves and others has uncovered the link between the immune system and the SCFAs in specific diseases such as autoimmune type 1 diabetes (T1D), obesity and type 2 diabetes (T2D), cardiovascular disease, infections, allergies, asthma, and IBD. Thus, the power of these three gut dynamic components—the mucosal immunity, the microbiota, and diet—can be harnessed in tandem for the prevention and treatment of many inflammatory and infectious diseases.

## Introduction

Considered the largest immunological organ in our body, the gut is in constant contact with food antigens, commensal microbiota, and foreign pathogens. As such, the gut has evolved extremely dynamic anatomy capable of regulating innate and adaptive immune responses to manage these interfaces effectively (1). In a homeostatic environment, there is a mutualistic relationship between the host and the microbiota. Keeping the delicate balance between mucosal immunity and microbiota (commensal bacteria) is a complex process. A disruption to the gut microbiota ecology, also known as dysbiosis, can lead or contribute to disease (2–4). While the host provides a nutrient-rich environment, the microbiota, in turn, provides the host with an increased capacity to harvest nutrients from food and boost the host immune system. The mucosal immune system, as a physical barrier, prevents pathogenic microorganisms and immunogenic components from traversing from the mucosae into the internal environment of the host. At the same time, this tightly regulated system has the important task of inducing tolerance to antigens (1). Here, we review the complex interplay between intestinal immunity, gut microbiota, and diet in inflammatory diseases (Table 1), such as T1D and IBD (22), and infection in humans and animal models.

**Table 1:** Diet, intestinal immunity and microbiota in diseases.

**Disease**	**Involvement of intestinal immunity and the gut microbiota**	**References**	
		**Animal models**	**Humans**
Type 1 diabetes (T1D)	• Limited or altered microbiota diversity	(5–12)	(13–21)
	• Increased gut permeability		
	• Intestinal immune system in a constant state of pro-inflammation		
Inflammatory bowel disease (IBD)	• Reduction of butyrate-producing bacteria/butyrate production	(22, 23)	(24, 25)
	• Tryptophan-rich diet and L.reuteri induced differentiation of TCRαβ^+^CD4^+^CD8αα^+^ IELs associated with protection	(26–29)	
	• Pathogenic role for TCRγδ^+^ IELs in colitis	(30, 31)	
Infections	• Absence of commensal microbiota regulated CD8αβ^+^ IELs associated with protection against bacterial and viral infections	(32–35)	
	• Crosstalk between IECs and TCRγδ^+^ IELs within the epithelium prevented pathogen invasion	(36–40)	

## Dynamic trio: intestinal immune system, gut microbiota, and diet

A network of intestinal immune cells resides within the epithelium and consists of three distinct lymphoid structures. This physical and biochemical barrier to commensal and pathogenic microorganisms is formed by the epithelium layer, the lamina propria layer (LPL) and the gut-associated lymphoid tissues (GALT). The GALT comprises the mesenteric lymph nodes (MLNs), the Peyer's patches (PPs) and isolated lymphoid follicles (ILFs) (1). Intestinal epithelial cells (IECs) have fundamental immuno-regulatory functions. For example, trans-presentation of IL-15 by IECs drives the development and differentiation of immuno-regulatory CD8αα intraepithelial lymphocytes (IELs) (41). Furthermore, overexpression of epithelial IL-15 is essential for the motility and localization of protective γδ IELs into the epithelium of small intestine (42). The IECs also express anti-inflammatory cytokines such as IL-10, which likely contributes to tolerance to commensal bacteria and epithelial integrity through epithelial-macrophage crosstalk (43). Besides that, epithelial cells are reinforced by tight junctions to form an interconnected network that acts as the main mechanical barrier of the intestinal mucosal surface. Tight junctions, such as zonulins, occludins and claudins, are essential for border control and are strictly regulated in response to various signals to preserve cellular polarity (44). Specialized IECs such as goblet and Paneth cells reinforce barrier function by the secretion of antimicrobial peptides (AMPs) by enterocytes (45). These AMPs, such as C-type lectin regenerating islet-derived protein IIIγ (REGIIIγ) and defensins, disrupt surface membranes of bacteria; thus enabling a broad regulation of commensal and pathogenic bacteria (46–48).

The intestinal innate immune system mediates the symbiotic relationship between the host and the gut microbiota. Myeloid differentiation primary response gene 88 (MyD88) is a central adaptor molecule for the majority of Toll-like receptors (TLRs) involved in pathogen recognition. To preserve symbiosis, IECs need to recognize pathogenic molecule patterns. Previous data support MyD88 playing a key role in regulating the immune system by shaping the gut microbiota (6). Furthermore, IEC MyD88 has been suggested to act as a primary sensor involved in the cross-talk between nutrients, the gut microbiota and the host during diet-induced obesity (DIO) (49). The specific deletion of IEC MyD88 partially protected against DIO, diabetes and inflammation. Previously, Ley et al. (50) discovered that genetically obese mice have a 50% reduction in the abundance of Bacteroidetes and a proportional increase in Firmicutes. In another study, the authors performed microbiota transplantation experiments and demonstrated that the obese microbiota had an increased capacity to harvest energy from the diet (51). Additionally, IECs are also involved in the adaptive immune regulation of gut homeostasis mediated by IgA (52). Another type of specialized IECs is concentrated in the follicle-associated epithelium which overlays the luminal surface of lymphoid structures including PPs and ILFs. These microfold cells or M cells facilitate the sampling of luminal antigens and intact microorganisms for presentation to the intestinal immune system (53, 54). While both M cells and goblet cells have been shown to be involved in antigen delivery to the LPL, their functional importance to and influence on the regulation of pathogen responses or immune tolerance remain incompletely identified.

The connections between the gut microbiota and the mucosal immunity are vastly influenced by dietary fiber and microbial SCFAs. Fermentation of different foods, such as indigestible polysaccharides by the gut micrbiota, produces SCFA acetate, propionate and butyrate (55, 56). For instance, *Bacteroides thetaiotaomicron*, an acetate producer, upregulated KLF4, which is involved in goblet cell differentiation and increased expression of mucin-related genes. *Faecalibacterium prausnitzii*, an acetate consumer and butyrate producer, attenuated the effects of *B*. *thetaiotaomicron* to prevent mucus overproduction, necessary for a healthier epithelium structure and composition (57). The gut epithelium is covered by a mucus layer and its integrity is vital to prevent microbe invasions and infections (1). As elucidated by Desai et al. (58), a chronic or intermittent dietary fiber deficiency caused the gut microbiota to resort to using host-secreted mucus glycoproteins as a nutrient source, leading to erosion of the colonic mucus barrier. The goblet cells secrete heavily glycosylated proteins called mucins into the intestinal lumen as a first line of defense against microbial invasion (45). Mucin 2 (MUC2), the most abundant of these mucins, is pivotal in shaping the intestinal mucus layers at epithelial surfaces. Mice deficient in MUC2 experienced fatal colitis when infected with *Citrobacter rodentium* (59). It is striking to observe that simple deprivation of dietary fiber in wild-type mice for either the whole duration or intermittently in the 40-day study period was sufficient to imitate the phenotype in MUC2-deficient mice. Furthermore, a prolonged lack of dietary fiber is associated with increased abundance of mucin-degrading bacteria such as *Akkermansia muciniphila* (58). Consistent with this, feeding mice a Western diet with very low fiber content has been shown to strongly affect gut microbiota composition, reduce mucus growth rate and increase penetrability of the inner colonic mucus layer (60). Given that fiber is the primary source of energy for the gut microbiota, the results ascribed to the above studies point to the critical effect of microbial SCFAs. As such, *Bifidobacterium* strains have long been used as probiotics, with several beneficial effects attributed in part to the increased production of acetate (61). For example, colonization of germ-free (GF) mice with bifidobacteria prevented translocation of enteropathogenic *Escherichia coli* O157 (61). Although supplementation of 1% inulin (a prebiotic with bifidogenic effect) or *Bifidobacterium longum* prevented mucus defects, it was insufficient to improve metabolic parameters of obese animals. Contrastingly, a high intake of 20% inulin reduced microbiota encroachment into the mucosa, prevented colonic atrophy and increased intestinal epithelial proliferation, thereby protecting mice against DIO (62).

## IELS: friend or foe in disease?

To sustain gut barrier integrity, the mucosal immune system is prepared to provide extra protection against infection and inflammation. Commonly referred to as intestinal intraepithelial lymphocytes (IELs) (63), most IELs express the CD8αα homodimer and approximately 90% express T cell receptors (TCRs) (64) and can be classified as induced (conventional) or natural (unconventional). Induced IELs are derived from peripheral T cells that home to the intestinal epithelium after encountering antigens in gut-associated lymphoid tissues (GALT). Induced IELs can be divided into subsets that express either CD4 or CD8αβ, upon entering the intestinal epithelium (63). Induced IELs typically exhibit an effector-memory-like phenotype and are thought to have a protective role against pathogenic invasions (63). A fraction of the induced TCRαβ^+^CD4^+^ T cells migrate to the intestinal epithelium upon activation in GALT where they can act as effector or tissue-resident memory T cells. While CD4^+^ T cells have been traditionally thought to lose the CD8 chains after thymic development, some studies have reported the existence of TCRαβ^+^CD4^+^ IELs that also express CD8αα both in mice (65, 66) and humans (67), which do not possess every quality of mature CD8^+^ T cells. In order to gain expression of CD8αα, mature CD4^+^ T cells need to reactivate CD8-lineage genes by terminating expression of T helper master regulator (ThPOK), while concurrently upregulating expression of the transcription factors Runx3 (68) and T-bet (69). External stimuli within the intestinal epithelium, for example, TGF-β, retinoic acid, IFN-γ, and IL-27, serve as cues for the re-differentiation of CD4^+^ T cells (69–71).

Recent data confirm the gut microbiota as yet another central factor in the generation of TCRαβ^+^CD4^+^CD8αα^+^ IELs (26). For example, a tryptophan metabolizing commensal microorganism called *Lactobacillus reuteri* has been revealed to activate the transcription factor aryl hydrocarbon receptor (AhR), important for immune regulation in mucosal inflammation. Tryptophan is mostly found in foods such as oats, dairy products, seeds and, nuts. Mice without *L. reuteri* have significantly low numbers of TCRαβ^+^CD4^+^CD8αα^+^ IELs, and reconstitution of *L. reuteri* in these mice, coupled with a diet rich in tryptophan, was adequate to induce TCRαβ^+^CD4^+^CD8αα^+^ IELs differentiation (26). This IEL subset has an immune regulatory effect in the prevention of IBD (27, 28). An elegant study by Sujino and colleagues further demonstrated a microbiota-dependent conversion of LPL Foxp3^+^ T_regs_ into TCRαβ^+^CD4^+^CD8αα^+^ IELs upon migration to the epithelium to control intestinal inflammation (29). TCRαβ^+^CD8αβ^+^ IELs are another type of induced IELs that make up 10-15% of the IEL population in mice (63) and 70–80% in humans (67). The majority of this subset is derived from peripherally activated CD8^+^ T cells that eventually migrate to the intestinal epithelium (72) and remain there. These cells have properties distinct to antigen-experienced CD8^+^ T cells found in peripheral lymphoid organs (73) in the expression of activation markers and cytokine secretion. The expression of CD8αα, which decreases the sensitivity of the TCR to antigens, is another main factor differentiating peripheral and intestinal CD8^+^ T cells (74). Presently, the role of TCRαβ^+^CD8αβ^+^ IELs in mucosal immunity is still unclear. Adoptive transfer studies have demonstrated a protective function of antigen-specific CD8αβ^+^ IELs in mouse models of bacterial and viral infections (32, 33). Although the CD8αβ^+^ IELs are strongly cytotoxic, they typically encounter commensal bacteria rather than invasive pathogenic microorganisms. Many studies comparing conventional, GF and antibiotic-treated mice and attempting to explore the connection between commensal bacteria and CD8αβ^+^ IELs have found a positive relationship, where the former may be involved in regulating the number of CD8αβ^+^ IELs in the intestinal epithelium (34, 35). Whilst that may be true, the mechanism behind this phenomenon is unknown. A recent study by Chen et al. (75) identified a series of genes that encode antimicrobial peptides, especially α-defensins, in commensal-dependent CD8αβ^+^ IELs using microarray analysis. Applying 16S rDNA sequencing techniques in their microbiota-transplantation experiment, the authors showed that depleting the *Bifidobacterium* species using antibiotics increased the number of CD8αβ^+^ IELs. They further demonstrated that the IL-15/IL-15R complex influenced by commensal bacteria enhanced the antimicrobial activity of CD8αβ^+^ IELs *in vitro*. However, the mechanistic link showing how a specific bacterial species or particular microbial metabolites could influence these IEL subsets remains unexplored.

While the gut immune system can replenish itself, interaction with the thymus is also essential to support gut homeostasis. Natural IELs such as TCRγδ^+^ cells migrate directly to the intestinal epithelium after development in the thymus (63). However, there is evidence to suggest the extrathymic origin of many TCRγδ^+^ IELs (76). TCRγδ^+^ cells are not only limited to the intestines, but have also been found in other mucosal sites such as the skin and uterus where they contribute to the maintenance of barrier integrity (77). These cells have been shown to have dynamic migratory patterns within the intestinal epithelium mediated by occludin, a tight junction protein (36, 37). We found that increased expression of occludin in the colon of non-obese diabetic (NOD) mice fed with a high butyrate-yielding diet was associated with protection against autoimmune diabetes (5). Remarkably, we found butyrate increased colonic TCRγδ^+^ IELs correlated to increased expression of IL-15 in the thymus (Y. Yap, J. L. Richards & E. Mariño, unpublished data). Epithelial IL-15 is essential for the migration of TCRγδ^+^ IELs into the intestinal epithelium (42). Additional *in vitro* analyses further supported the importance of epithelial IL-15 and IL-2Rβ in TCRγδ^+^ IEL motility within the intestinal mucosa. Impaired IL-2Rβ signaling and overproduction of IL-15 locally in the lamina propria resulted in acute pathogenic *Salmonella typhimurium* invasion and enhanced bacterial translocation (42). A fundamental role of TCRγδ^+^ IELs is to protect the intestinal epithelium against pathogenic microorganisms and inflammation. Crucial in the mucosal immune response against resident intestinal bacteria, TCRγδ^+^ IELs produce antimicrobial effectors such as RegIIIγ when in contact with resident bacterial pathobiont within the IECs (38). This antibacterial response is dependent on MyD88 signaling in IECs, which further highlights the microbe-dependent cues for TCRγδ^+^ IEL homeostasis. This crosstalk between IECs and TCRγδ^+^ IELs is not only crucial for intestinal homeostasis with resident microbiota but also necessary in the protection against pathogens, such as *S. typhimurium* (38), *Toxoplasma gondii* (39) or the parasitic *Nippostrongylus brasiliensis* (40). The function of TCRγδ^+^ IELs may differ between the small intestine and colon and may be regulated by different mechanisms depending on the presence of injury to mucosal tissues (38, 78, 79). To protect the epithelium against inflammatory damage during infection, TCRγδ^+^ IELs produce a variety of factors such as TGF-β and keratinocyte growth factor (KGF) to promote healing and integrity of the intestinal epithelium (80).

The cytotoxic and repairing properties of TCRγδ^+^ IELs is evident in infections by fighting off early microbial invasion and limiting excessive tissue damage at the end of the infection phase. In experimental models of colitis, TCRγδ^+^ IELs appear to have diverging roles. Some studies have demonstrated a pathogenic role for these cells in colitis induction (30, 31). Interestingly, as the disease progressed to its later stages, TCRγδ^+^ IELs play a protective role against inflammation-induced epithelium damage (81, 82). Mice lacking cytosolic sensor for microbial DNA, STING, are highly susceptible to colitis and acute infection (83). Exacerbated colonic inflammation correlated with a significantly higher frequency of natural TCRγδ^+^ IELs, decreased induced TCRαβ^+^CD8αβ^+^ and TCRαβ^+^CD8αα^+^ IELs and impaired development and function of Foxp3^+^ T_regs_. As such, these findings strongly suggest the highly variable impact of the microbiota on the frequency and function of intestinal IELs in infection settings. Intestinal IELs have largely been studied in disease settings where they are involved in mechanistic links with the gut microbiota. In fact, mice were protected from ischemic stroke when intestinal homeostasis was restored (84). Mice induced with acute brain injury presented an altered gut microbiota, which promote the trafficking of effector IL-17-producing TCRγδ^+^ cells from the small intestine to brain. Protection against ischemic stroke was correlated with the suppression of effector TCRγδ^+^ cells (84). In autoimmune diabetes, TCRγδ^+^ cells, depending on their origin and condition, can either protect (85–87) or exacerbate (7) the disease, once again highlighting the dual role of these cells in disease.

## Dietary factors in action

Environmental factors are dominant over host genetics in shaping the human gut microbiota (88). As we have shown, diet is one environmental factor that highly influences the gut microbiota and the immune system (5, 22, 89, 90). Diet is composed of different macro- and micronutrients, and natural sources of fiber are necessary for the production of SCFAs (56). Conversely, changes in our dietary habits to include highly processed and refined foods, additives such as emulsifiers and preservatives, can negatively impact on gut microbial composition, which may adversely affect our health (91–94). Acetate, propionate and butyrate contribute to a healthy gut microbiota and for a mature and developed immune system (55). We have shown in a mouse model of colitis, the SCFAs, acetate and butyrate protected against intestinal epithelial damage in DSS-induced colitis (22). High fiber feeding promoted the release of SCFAs, which led to inflammasome activation through GPR43 and GPR109A (22). This event led to the production and secretion of IL-18, a cytokine involved in repairing intestinal epithelial integrity, and homeostasis. The high levels of IL-18 and protection from DSS colitis were associated with a significant increase in *Bacteroidetes*, which were predominantly found in mice fed a high fiber diet. Conversely, species that were considerably low or absent in mice fed a zero-fiber diet were bacteria from the genus *Prevotella*, known to contain a set of bacterial genes for cellulose and xylan hydrolysis. This is consistent with findings in children from rural Burkina Faso study, whose microbial diversity is associated with the extraction of valuable nutrition from fibrous plant foods (95, 96).

SCFAs also regulate the immune cells that reside in the LPL. As such tolerogenic mucosal CD103^+^ dendritic cells (DC) conferred protection in a murine food allergy model through high fiber feeding. In this study, we found a high fiber diet increased the release of acetate and butyrate associated with enhanced oral tolerance and retinal dehydrogenase (RALDH) activity (90). Furthermore, protection from food allergy was dependent on vitamin A found in the diet. Accordingly, mice lacking GPR43 or GPR109A (metabolite sensing receptors for acetate and butyrate) displayed exacerbated food allergy and reduced CD103^+^ DCs. Likewise, butyrate produced by commensal bacteria promotes peripheral T_reg_ generation via inhibition of histone deacetylases (HDAC) inhibition (97) or the induction of tolerogenic DCs (90) in mice. Similarly, *in vitro* studies show butyrate to be a potent inducer of human tolerogenic DCs by inducing RALDH activity and type 1 regulatory T cells (Tr1) differentiation via simultaneous HDAC inhibition and signaling through GPR109A (98). Previous gut microbiome analysis revealed a significant reduction in the number of butyrate-producing bacteria, such as *Firmicutes* and *Lachnospiraceae* in the colon of patients with IBD (24) and colorectal cancer (25). The gut microbiota through GPR109A signaling have anti-inflammatory properties by promoting colonic macrophages and DCs to induce differentiation of T_reg_ cells and immune-regulatory IL-10-producing T cells (23). GPR109A was also essential to induce IL-18 via a butyrate-mediated pathway in the colonic epithelium for intestinal integrity. Besides butyrate, GPR109A also acts as a receptor for niacin (or vitamin B3), which is also produced by the gut microbiota. Accordingly, niacin-deficient (*Niacr1*^−/−^) mice displayed exacerbated DSS-induced colitis pathogenesis. Complete ablation of the gut microbiota or depletion of dietary fiber greatly increased the risk for DSS-induced colitis and colon cancer in mice (23). As previously discussed, there is a duality in the role of IELs in infection. We and others have found that a high acetate-yielding diet reduced colonic atrophy and inflammation in mice affected with *C. rodentium* infection in a GPR43-dependent manner (99) (K. H. McLeod, Y. Yap & E. Mariño, paper under review). SCFAs mediated by GPCRs conditioned the IECs to produce inflammatory cytokines and chemokines such as IL-6, CXCL1 and CXCL10 for a timely response against bacterial products in the protection against infection; thus highlighting again the critical connections between intestinal immunity, the gut microbiota and diet (99).

As previously mentioned, the SCFAs can exert their anti-inflammatory properties through HDAC inhibition (97, 100, 101). We have shown that feeding mice a high fiber diet enhanced T_reg_ numbers and function, which protected them against allergic airways disease (AAD) (89). In that study, we demonstrate that dietary acetate inhibited HDAC9 by increasing acetylation at the Foxp3 promoter, which led to increased T_reg_ cells (89). Further analyses demonstrated an increase in fecal and serum SCFA concentrations, particularly acetate, and identified the dominance of an acetate-producing bacteria, *Bacteroides acidifaciens* A40(T) strain in mice fed a high fiber diet. Likewise, another study reported that dietary fiber and the SCFA propionate protected against AAD in mice (102) in a GPR41-dependent manner. Dietary fiber decreased the ratio of *Firmicutes* to *Bacteroidetes*. Furthermore, mice treated with propionate had alterations in bone marrow hematopoiesis marked by enhanced generation of macrophage and dendritic cell (DC) precursors with impaired Th2 response in the lung. We observed in both mice and humans that a higher intake of dietary fiber during pregnancy was associated with a higher concentration of serum acetate and increased protection against the development of airway disease in offspring (89).

## Putting the brakes on autoimmune T1D

T1D is one of the most common autoimmune diseases that results in specific T cell-mediated destruction of pancreatic β-cells in genetically susceptible individuals (103). Although most of the action occurs in the pancreas and adjacent lymph nodes, the gut has been implicated as an early regulator of disease progression (104, 105). In fact, the progressive rise in global T1D incidence (106, 107) points to environmental factors, such as infections, antibiotics and diet, having a great impact on the development of T1D (94, 108–110). We believe that studying the synergistic roles of the intestinal barrier, the microbiota composition and the mucosal immune system offer potential intervention targets to treat T1D. Evidence shows that NOD mice (5, 8–10) and children diagnosed with T1D have a distinct and limited gut microbiota diversity (13), with an increased ratio of *Bacteroidetes* to *Firmicutes*, compared to healthy individuals (14). Furthermore, metagenomic data showed a significant reduction in abundance of butyrate producers, such as *Faecalibacterium*, and mucin degraders, such as *Prevotella* and *Akkermansia* (15, 16). This is in contrast with other SCFA producers such as *Bacteroides* and *Alistipes*, which were more abundant in the feces of diabetic individuals (15). As mentioned previously, butyrate is central for epithelial integrity, therefore an altered gut microbiota composition may contribute to a “leaky” gut and aberrant intestinal immune system, all contributing to the etiology of T1D.

In T1D, increased gut permeability contributes to intestinal inflammation, shown in both animal models (11) and human patients (17). Likewise, intestinal infection that promote a “leaky” gut also promoted accelerated T1D in mice by increased activation and proliferation of diabetogenic CD8^+^ T cells in the pancreatic lymph nodes (PLNs) (111). The role of the gut in the loss of tolerance to self-antigens that leads to autoimmune T1D is poorly understood. However, an unbalanced host-microbe interaction and excess of food and microbial antigens may skew the intestinal immune system toward a perpetually pro-inflammatory state (12). Likewise, children with T1D show overall increased intestinal immune activation by high IL-4, IL-1α and IFN-γ protein expression in the small intestine LPL (18). The number of regulatory Foxp3^+^ T_regs_ are reduced and/or inactive in biopsied small intestine tissues in T1D patients (19). Moreover, it has been reported that children newly diagnosed with T1D had reduced Foxp3^+^ T_regs_ and increased IL-17-expressing CD4^+^ and CD8^+^ T cells in the blood, indicating the absence of immune suppression mechanisms (20). In contrast, adoptive transfer of splenic IL-17-producing TCRγδ^+^ cells did not exacerbate diabetes in NOD mice, but instead had regulatory effects through TGF-β upregulation (87). Inducing mucosal immune tolerance through aerosolized insulin has been considered a therapeutic approach to treat T1D in secondary prevention studies. Interestingly, transfer of purified splenic CD8^+^γδ T cells from diabetic NOD mice treated with insulin aerosol suppressed diabetes. Protection was associated with reduced ${\text{insulin}}_{\text{B}9-23}^{+}$CD8^+^ T cells, increased IL-4 and IL-10 cytokines and reduced GAD^+^CD4^+^ T cell proliferation (85). This study magnifies the role of the gut mucosal immunity in the development of T1D. As such, an impaired thymus, the site for T cell selection and maturation including the IELs, accelerates autoimmune diabetes in mice. Remarkably, transfer of purified TCRγδ^+^CD8αα^+^ IELs from NOD mice prevented diabetes by reconstituting the small intestine IELs compartment of recipient mice that have undergone neonatal thymectomy (86). There is however, evidence to suggest a pathogenic role for TCRγδ^+^ cells in T1D, as adoptive transfer of a specific subset (CD27^−^TCRγδ^+^) of these cells into NOD.SCID recipients accelerated T1D development. Besides, knockdown of the TCRδ receptor in NOD mice offered robust T1D protection (7). As the studies above have shown, there is more than meets the eye when it comes to the role of TCRγδ^+^ cells in the story of T1D pathogenesis.

Our research group has focused on new insights studying the connectivity between the gut microbiota, mucosal immune system and diet for the prevention and cure of T1D. We were the first ones to elucidate that an altered gut microbiota and reduced production of SCFAs are signatures of T1D in NOD mice as it has been recently confirmed in individuals with T1D (21). We showed that exacerbated immune responses against islet antigens and increased gut leakiness were ameliorated by specifically augmenting the delivery of dietary acetate and butyrate. Therefore, we use a dietary intervention as an alternative therapeutic approach to halt immune-mediated beta-cell damage, promote a balanced and healthy gut microbiota, and abrogate the progression to clinical diabetes. We demonstrated that high SCFAs-yielding diets protected NOD mice from autoimmune diabetes (5). Acetate regulated autoreactive CD8^+^ T cells through changes in B cell differentiation and function. On the other hand, butyrate enhanced splenic and colonic Foxp3^+^ T_reg_ cell numbers through histone modification. Moreover, the diets high in acetate and butyrate significantly restored gut epithelial integrity by reducing circulating bacterial lipopolysaccharide (LPS) concentration, correlated an with increased expression of occludin. Recently we have shown, consistent with the previous results, that high butyrate-yielding diets increased the number of colonic TCRγδ^+^ IELs associated with increased thymic IEL precursors (Y. Yap & E. Mariño, unpublished data). Overall, our results demonstrated the astonishingly beneficial effects of high SCFA-yielding diets, by targeting both the gut microbiota and the immune system, thus offering a promising outcome on the prevention and treatment of autoimmune and inflammatory diseases.

Whilst supplementation of dietary SCFAs proved to be a safe and non-invasive therapy in a mouse model of T1D (5), one cannot help but wonder if using dietary supplementation together with current pharmaceutical approaches will favorably and greatly increase treatment efficacy. Indeed, therapies combining dietary treatment with immunotherapy are now starting to be tested in various disease models. For example, a very recent study testing the combination of a ketogenic diet and PI3K inhibitors, such as metformin and SGLT2, demonstrated significantly enhanced efficacy/toxicity ratios in various murine models of cancer (112). If these findings were successfully, translation into human clinical trials may revolutionize our current therapeutic strategies to eventually pair dietary or medicinal food supplementation with targeted immunotherapy for the treatment of many autoimmune and inflammatory diseases.

## Concluding remarks

Altogether, these studies provide validation that the impact of dietary metabolites within the gut has local as well as far-reaching anti-inflammatory effects on host health. Taken together, these findings accentuate the powerful impact the triage of intestinal immune health, the gut microbiota and dietary factors have in the pathogenesis of various autoimmune and inflammatory diseases such as T1D, infections and IBD. Much effort has been made in recent years to uncover the exact mechanisms behind their effects on health and disease. We are advancing on sophisticated methods such as genomics, transcriptomics, proteomics, metabolomics and nutrition that allow us to understand how this dynamic trio works together. While more studies in humans are required, dietary SCFAs have shown to be promising novel therapeutics. Dietary SCFAs therapy may be specifically tailored and designed, alone or in combination with potential immunotherapies, to prevent or cure diseases, where the future of medicine lies.

## Author contributions

All authors listed have made a substantial, direct and intellectual contribution to the work, and approved it for publication.

### Conflict of interest statement

The authors declare that the research was conducted in the absence of any commercial or financial relationships that could be construed as a potential conflict of interest.
